# Caregiver Knowledge of Long-Term Services and Supports: Effects of Rurality and Support

**DOI:** 10.1093/geroni/igaa057.1146

**Published:** 2020-12-16

**Authors:** Lauren Stratton, Nichole Richter, Mack Shelley, Jennifer Margrett

**Affiliations:** Iowa State University, Ames, Iowa, United States

## Abstract

Caregivers often lack knowledge regarding available long-term services and supports (LTSS). Certain barriers, such as rurality and levels of social support, may contribute to a lack of knowledge and accessibility of LTSS. The Caregiver Beginnings Workshop, held in 12 communities throughout 11 counties in Iowa, was a one-time educational session created to increase knowledge and awareness of LTSS. Data were collected from pre- and post-tests completed during the workshop (N = 98). To assess caregivers’ initial knowledge of LTSS, a hierarchical regression model was estimated to examine knowledge of LTSS in caregivers as predicted by caregiver education, number of health problems in care recipient, relationship type, feelings of social support, and rurality. Results showed that rurality (β = 0.33, p = 0.047) and infrequent or no support (β = -0.30, p = 0.02) were significant predictors (R2 = 0.21), indicating that caregivers living in rural areas reported higher knowledge of LTSS and those who reported infrequent or no support reported less knowledge. Additional analyses examined county-level data to better understand the availability of community resources in rural areas. County-level variables (e.g., number of home healthcare services, education level, income, health status) were included in a regression model to predict knowledge of LTSS. The results indicated that median income (β = -0.32, p = 0.002) and an educational attainment of an associate’s level degree or higher (β = -0.30, p = 0.004) were significant predictors. Discussion focuses on the importance of support and accessible resources for caregivers in all geographic areas.

